# Report of Surgical Cases at the Buffalo General Hospital

**Published:** 1862-10

**Authors:** J. R. Lothrop

**Affiliations:** Attending Surgeon


BUFFALO

### Medical and Surgical Journal

VOL. II. OCTOBER, 1862. NO. 3.

### ORIGINAL COMMUNICATIONS.

ART. I.—*Report of Surgical Cases at the Buffalo General Hospital. By* J. R. LOTHROP, *Attending Surgeon.*

14.—The four instances of paralysis were cases of true spinal paralysis or paraplegia, involving a more or less complete loss of power in the lower extremities, with impaired sensation in some, but not in all. One was traumatic, one, as was rather conjectured than positively ascertained, depended upon disease of the bones, and in the others no definite cause could be assigned, though the slow progress and other symptoms led to the suspicion of softening in one of them.

The case of traumatic paralysis was caused by gun-shot wound. Three years before, the patient had been shot in the back by a rifle bullet, it entering about three inches from the spinal column, opposite the lower dorsal region and passing obliquely across the body, leaving no wound of exit. From the result it seemed probable that it lodged in the spinal column. The symptoms in this case were, first, loss of motion not entire, for the patient when recumbent could draw up the legs, but to such an extent that they were powerless for walking, or even sustaining the weight of the body. Sensation was somewhat impaired. There was a flabby state of the abdominal muscles, constipation obstinate and continued, generally great distension of the intestines with flatus, pains in the limbs and loss of power in the sphincter ani, so that liquid matter passed involuntarily, but the solid contents were retained. There was in some degree both incontinence and retention of urine—incontinence was most marked; the urine, moreover, had that marked phosphatic character so often seen in injuries of the spinal cord, the deposit being abundant enough to coat thickly the vessel containing it.

The second case in which it was supposed that the cause was disease of the bones, exhibited mainly loss of motor power in the lower extremities, but with no impairment of sensation, no loss of control of the sphincters, no constipation, and no pains or abnormal sensations in the limbs themselves. The loss of motor power in this case was not entire, as the power of moving the limbs slightly when in the recumbent posture was retained, and also of sustaining the weight of the body for a short time, but no power to move forwards. The patient was therefore enabled to use crutches, dragging the limbs after him.

In one other case the paralysis was of slow development, affecting both motion and sensation; the interference with the latter did not amount to anæsthesia, but rather numbness, or the sense of creeping of ants. There was also a pendulous state of the abdominal muscles, constipation and retention of urine. In this case also the urine was alcaline.

As has been stated these three cases in which the attempt to diagnosticate the seat and nature of the spinal affection, were thought by the differences in the symptoms to indicate, in the first, an affection of the membranes of the cord in the lower dorsal region, producing pressure, but no interruption of the continuity of the cord; in the second, disease of the bones in the lower part of the spinal column, causing loss of motion as the main symptom, though there was an absence of local symptoms, such as pain, tenderness or prominence in any part of the column; in the third, softening of the cord, evidenced by long continued, slowly progressing loss of power and sensation, with abnormal sensibility, such as numbness and formication.

This attempt at differential diagnosis is always a matter of more or less difficulty. But some aid may be derived from a careful study of the symptoms. The following considerations assist in the determination of the peculiar nature and seat of the disease. Applied to the cases above described, the ground upon which the particular diagnosis was made will be apparent.

As to the seat of the disease or injury, as far as it has any bearing upon the above cases, it is enough to confine our attention to the lower dorsal and lumbar portions of the spinal column. Affections of the lower dorsal region are attended by a relaxed and flabby condition of the abdominal muscles, so as to render the abdomen pendulous, a state often observed in spinal paralysis. The loss of compressing power in these muscles also favors and causes confirmed constipation, and also the accumulation of flatus to a great extent. This loss of compression is one of the conditions necessary for great accumulation of urine in the paralyzed bladder, which cannot take place as long as they can exert a power to force the urine out.

Injuries to and diseases of the lumbar region, and of the sacrum, besides causing paralysis of the lower extremities affect the pelvic muscles and organs without causing loss of power in the abdominal muscles. Thus the bladder may be affected with both retention and incontinence, a certain accumulation being always present, but incontinence being evident by a constant overflow. This accumulation must be limited as long as the abdominal muscles retain their power of compression.

The symptoms may aid us in determining the nature of the disease. Affections of the bones frequently involve the motor functions, but leave the power of the sphincters unimpaired; thus we may meet with cases in which there is entire motor paralysis of the lower limbs, with perfect control of both the sphincter of the bladder and anus. Apart from the local symptoms which are more distinct in affections of the bones than in any other spinal disease—they afford some aid. Usually there is alteration of shape, or tenderness at the seat of the disease of the bones in spinal paralysis, but it may not always be distinct.

Diseases of the membranes of the cord with thickening, and morbid growths which cause pressure upon it, but do not affect the continuity of its fibres, in addition to the loss of motion and sensation, which may be and often is incomplete, cause pains in the affected limbs and often spasmodic contractions.

Softening of the cord being generally slow in its progress, the paralysis resulting is for a long time incomplete. It may amount to only a slight diminution of power and perverted sensibility rather than pain, numbness being the most common form of impaired sensation, and slight uncertainty or tripping in the gait, of impaired motor conduction. Sudden paraplegia is a very rare result of disease, though frequently of injury. When unconnected with injury, it is caused by hæmorrhage, and attended with great pain in the spinal cord.

In the treatment of spinal paralysis, as the result of disease, the experience of most has probably convinced them of the hopeless nature of many of the cases which come under notice. Paralysis arising from diseases of the bones admits of the most certain improvement both temporary and permanent. Certain and marked benefits are often observed to follow rest and counter-irritation. But in paralyzed states, known to depend upon some morbid condition of the cord, or its membranes, as softening, chronic inflammation, effusion or exudation, it is hardly necessary to recall to many how often all measures have entirely failed. Counter-irritation, strychnia, electricity, and other means, though in turn to be tried, have proved entirely useless, and any treatment which may be proposed will too often disappoint us in the amount of its benefit. At the same time through the slow restoration of Nature, recovery sometimes takes place. The paralysis of the muscles, which results from atrophy and disuse, after the central affection has disappeared, is more within the reach of curative means. General measures to increase their nutrition, among which are friction and forced use, are attended with success.

15.—The three cases of erysipelas were, one of the head and face, one of the arm, traumatic, and one of the lower extremities and lower part of the body, attended with a fatal result.

The first and last occurred at the same time, and had their origin within the hospital; the third occurred at the same time with the others, but originated without. They all arose during the damp and cold weather of spring. There was nothing in their history or origin to give rise to the idea of contagion. The two arising in the hospital were in different wards.

The first, a case of erysipelas of the head and face, terminated as such cases mostly do in recovery, though it was of great severity. The treatment which appeared of most benefit was the external and internal use of the tinct. ferri muriat. This substance was used early, and the case went on very favorably, but a suspension of its use was followed by an increase in swelling and fever, marked enough to appear as a direct result of its discontinuance.

The second followed a fall upon the elbow, and involved the fore-arm mainly, the swelling being great. As the injured portion sloughed poultices enveloping the whole arm were used. Under their use the slough separated and the erysipelatous inflammation subsided.

The third was more extensive in its range. It began on one foot, at a point where there had been considerable pain for some time previously. This patient had suffered from frost-bite two years before, and had lost parts of both feet, partly by Nature's and partly by the surgeon's amputation. The feet, however, were painful at times after, and there was an occasional discharge of pus in the tarsal region, indicating a diseased condition of the tarsal bones. The swelling and redness began in the tarsal region, rapidly extended up the leg and thigh to the body, and crossing it passed down the other limb to the foot. It extended also up the back nearly to the shoulder blades behind, and the epigastrium in front. The swelling was very great, the progress of the affection rapid, and the constitutional depression very severe, so that the case terminated fatally in about a week. In the outset, while the disease was confined to one limb, cold applications were made, consisting of whiskey and cold water, but they did not arrest the progress of the inflammatory swelling. Moreover, as is sometimes observed in similar cases, they caused such an uncomfortable sense of chilliness, that their use was soon abandoned. Tincture of iodine was afterwards employed, but with no apparent influence to check the progress of the disease. Quinine and stimulants were freely given. This case was remarkable for the extent of the disease. When so large a portion of the body was involved, the fatal result is not surprising.

16.—The cases of syphilis were—three primary, three secondary, and five tertiary.

The cases of primary syphilis were not treated by the administration of mercury while in the hospital, though one had taken a full course of the proto-iodide of mercury before admission, at which time the gums gave evidence of its full influence. This patient was a female, and while in the hospital, some five or six weeks, exhibited no signs of secondary affection. The chancre had healed, and there was no glandular induration in the groin. While in the ward this patient having in her possession a box of pills which she brought in, each of which contained one grain of the proto-iodide of mercury, took at one time, of her own will, five of them as a cathartic. They did not produce a cathartic effect, but caused a most violent cramp and pain, recurring at intervals for two days, and followed by well marked peritonitis, which put her life in peril. When she recovered she left the hospital, and was not again under observation.

The other two cases were not treated by mercury, one because the chancre appeared to be of the soft or non-infecting variety—the other because it had given rise to gangrene of the prepuce, most of which was destroyed. The destruction had thoroughly removed the sore, whether infecting or not, and in the absence of any positive sign of constitutional infection mercury was withheld. The probabilities were all on the side of the sore's being a non-infecting one, as soft chancre is more likely to become gangrenous. The penis at one time exhibited a black encircling band, an inch broad, which upon separation left the gland unaffected, but decidedly uncovered. This young man remained under observation nearly two months, in which time no signs of secondary affection occurred.

The three cases of secondary affection were in the form of mucous tubercle about the anus and scrotum; in two cases the patch of raised granulations about the anus were three inches in diameter. The time from the primary infection varied from six to ten weeks. In these cases mercury was exhibited; in one case the blue mass three grains at night, in the other one grain of proto-iodide of mercury at night. The local treatment was a wash of dilute chlorinated soda, morning and night, and when dry, dusting with calomel. Under this treatment the disease disappeared in about three weeks.

The cases of teritary presented the varied and usual phenomena, but two were remarkable, one for the extent and severity of the infection, the other for the length of time which had elapsed since the primary infection. The first was a case of ulceration, affecting the body extensively, the sores being some of the deep, sloughy character, and others more superficial, and tending to spread in circles, with a partially healed centre. On some of the sores, when undisturbed, the thick, prominent, black crusts of rupia formed. This case was treated by tonics, various forms of mercury, as the bichloride and the iodides, by the iodide of potash, and by a combination of both, but the course of the disease was disastrous, and seemed likely to terminate in death. Syphilis does not inevitably tend to death, there being a tendency to elimination in many cases which go on without treatment.

The second case continued with intervals of tolerable health during thirteen years. Latterly the health of the patient was more firm. The traces of the disease were evident in cicatrices on various parts of the body. The remaining trouble was exfoliation of the bones of the palate, which was cleft nearly to the upper incisors, the soft parts being ulcerated, and portions of bone appearing in the margin. The whole of the back of the pharynx, with the openings of the Eustachian tubes was exposed to sight. This case was subjected to general tonic treatment, and improved while in the hospital, the ulcerations, being recent, healing, and the throat assuming a more healthy aspect.

It seems appropriate here to remark upon the giving of mercury in primary syphilis. Admitting two species of chancre the infecting and the noninfecting—the one always and the other never followed by constitutional syphilis—the necessity for the giving of mercury is limited to the infecting species alone. In the case then of an infecting chancre, what is the object aimed at in the use of mercury? Is it to promote the healing of the sore, or to prevent the occurrence of constitutional syphilis? Quite likely it may be given to secure both objects. But it is worth while to consider whether either is attainable by the means, and if not, to consider whether the use of so powerful an agent is advisable for a purpose beyond our power. The question is yet an open one. There seems to be a belief in the efficacy of mercury to promote the healing of the primary sore, and if it does, a careful employment of it is not altogether unadvisable. On the other hand statements are not wanting of careful observers that secondary symptoms appear by a law of disease, as invariable as the law governing the appearance of the eruption in scarlet fever, i. e. that they belong to the essential phenomena of the disease. In this view the giving of mercury is plainly altogether unnecessary and useless. Until more certain observations establish the impossibility of arresting the appearance of secondary symptoms in syphilis by the use of mercury, the practice will probably and wisely favor it The investigations which have been industriously made by intelligent and honest observers, incline us to consider the question as one yet subjudice. The above remarks apply to the giving of mercury to *prevent* the appearance of constitutional syphilis. When the secondary phenomena have appeared mercury is not only *a* proper, but so far as we know, *the* proper remedy to call to our aid.

17.—The two cases of malignant tumor were, one of schirrous affection of the breast and axilla in a male, with a most remarkable dissemination in the skin of the neighboring parts; the other, one of medullary tumor of the neck, just behind the angle of the jaw.

The first occurred in a man about sixty years of age, and was of several years' duration. He had a most cachectic appearance, as the disease was far advanced, and died a short time after the beginning of my term of service. The right breast was hardened, nodose. and drawn in, and a line of contraction and induration of glands extended to the axilla. In the axilla were several large, rounded, very hard masses, prominent and ulcerated. The largest separate masses were an inch in diameter. In the neighborhood, in the skin of the neck, breast and shoulder, were nodules, distinct and of various sizes, from that of a millet-seed to that of a common sized bean, movable on the texture beneath. The masses in the axilla partly by pressure and partly by the induration of the tissues, interfered with the circulation of the arm; in consequence it became enormously swollen and ædematous. This patient exhibited that fragility of the bones which is often found in the cancerous cachexia. About two months before his death he had a fracture of the femur, caused by stumbling over a chair. The broken bones had apparently firmly united at the time of his death. Just before death respiration was difficult, either by extension of the disease to the lung, or from effusion into the cavity of the pleura, which was more probable, as a marked dullness existed on the affected side.

The masses in the axilla were considered to belong to the variety of schirrus, as they were fixed, and of stony hardness; the hardened tumors in the breast also were of the same species, being fixed, and the skin drawn in over them, so that they were at the bottom of a depression. But the nodules in the skin being loose and isolated, need not necessarily be classed with the main growths, as medullary secondary formations in the skin are found accompanying large cancerous masses of primary formation.—Rokitansky, in speaking of fibrous cancerous nodules in the skin says, they are very often depressed beneath the surface of the skin, usually single, *firmly fixed*, and hard; and are generally primary cancerous growths, being the first of a series of cancerous formations in different organs of the body. They are generally confined to the face, though they may appear on other parts. Of the medullary secondary formations in the skin, he says, they are associated with large cancerous growths, and are scattered over large tracts of the body, especially the trunk. They grow in isolated nodules, near the primary mass, or they may appear in the skin after cancer has been localized in one or several organs, being then part of an extensive or general cancer production. They mark the general cancerous cachexia. In this case the brittleness of the bone furnished another indication of the extent of the cancerous tendency.

The second case occurred in a woman about thirty-five years of age, mother of several children, the youngest being an infant. The tumor was located in the space behind the angle of the jaw, which it filled, passed very slightly under the anterior border of the sterno-mastoid muscle, and overlapped to a small extent the parotid gland. Its growth had been rapid, and the health had been until shortly before quite good. At the time of admission the patient had the appearance of health, and made complaint only of pain in one of the thighs, and a slight lameness, which did not attract much attention. The tumor was rather larger than a hen's egg. quite prominent and pointed, movable and of moderate hardness.—The skin had a dark purplish hue over the tumor. As the patient desired the removal of the tumor, it was decided after consultation with my colleagues, to remove it. It appeared to be a loosely attached tumor, perhaps of glandular character, such as are found in that situation, and seemed likely to offer but little trouble in the removal, if indeed it might not be easily turned out. The dark color was the only circumstance which raised any suspicion of its being a malignant growth. The operation for its removal was made about the middle of March, the patient being under the influence of ether. After the first incision it was very evident it could not be turned out, but must be dissected with some care. Its removal by dissection was soon accomplished, and followed by but slight hæmorrhage. A small portion of the parotid was removed with it, as it had pushed into its substance slightly. The wound soon assumed a healthy appearance, and perfectly closed, though the cicatrization was not complete when the patient left the hospital, in about ten days.

The subsequent history of this patient shows most decidedly the tendency to general production of cancer. After her return home, the lameness of which she complained when in the hospital, and which was more important than it seemed, increased to such a degree that she was compelled to keep at rest. After a slight fall the thigh was fractured at the neck, and drawn up on the ilium, and any attempt to move it caused intolerable pain. A tumor at about the same time appeared in the right side of the abdomen, which enlarged rapidly, and occupied a large portion of the cavity of the abdomen, extending as high as the liver. This was found to be a large melanotic tumor, having but slight attachment with the mesentery.

The woman died about four months after the operation for the removal of the tumor from the neck. During the latter part of her life she was seen by Dr. Abbott of White's Corners, from whom the facts relating to her after she left the hospital were obtained.

The tumor, after its removal, was found to have no well defined sac which could be easily separated from the surrounding tissues. Although it might have seemed at first to have grown independently and merely pushed aside the neighboring textures; it in fact had penetrated them to a slight extent at its circumference, as well as pushed them aside. This was evident from the impossibility of dissecting it out clearly without injury to the textures in the vicinity. When divided through its centre its cut surface presented a variegated appearance, being divided into lobules of different colors sharply defined, several being very distinctly melanotic in hue; the others being variously reddish, yellow, or brownish yellow. The tumor was not very hard, and the lobular contents had a cheesy consistence.

The microscopic appearances were, 1st: Large cells, having one large oval nucleus containing several nucleoli. These cells occasionally exhibited a prolongation or process. 2d: Multinuclear cells somewhat misshapen, the nuclei often collected together at one side of the cell. 3d: Cells which appeared to be a mere agglomeration of fat granules, being in a state of fatty degeneration. 4th: Brownish granules of pigment, either enclosed within the cell walls or free. In a specimen of the large abdominal tumor given me by Dr. Abbot, the only distinct appearances were large cells with oval nuclei, and an abundance of brownish pigment granules and blood globules. The secondary formation in this case was not at the wound made by the removal of the primary tumor. It grew with remarkable rapidity in the abdomen. It belonged to the class of cancer melanodes, but the entrance of pigment into any cancer gives it the name of melanosis. Its entrance is most frequent in the medullary cancer. According to Rokitansky, in most cases melanosis is merely medullary carcinoma modified by pigment. The secondary tumor was in this case a rapidly growing medullary tumor, colored by the melanotic matter. In this case as in most whenever medullary cancer recurs, it assumes the most marked form, namely, that which is known as encephaloid. The history of this case adds confirmation to the infrequency of benefit resulting from operative interference with cancerous growths. Rare as is spontaneous cure in such cases, it is unfortunately hardly more rare than cure by surgical means.

